# SG-APSIC1083: Prevalence and classification of carbapenemase-producing gram-negative bacilli at a medical center in Ho Chi Minh City, Vietnam

**DOI:** 10.1017/ash.2023.81

**Published:** 2023-03-16

**Authors:** Tuan Huynh, Loan Luong

**Affiliations:** University Medical Center, Ho Chi Minh City, Vietnam; University Medical Center, Ho Chi Minh City, Vietnam

## Abstract

**Objectives:** The identification and classification of carbapenemases are meaningful in clinical treatment, epidemiology, and multidrug-resistant bacteria control. We sought to identify the proportion of carbapenemase-producing and carbapenemase classifications in gram-negative bacilli in our hospital. **Methods:** Isolates of gram-negative bacilli were extracted from sputum, blood, and urine samples in a medical center in Ho Chi Minh City. The identification of gram-negative bacilli was performed using the Phoenix M50 automated system (Becton Dickinson, Franklin Lakes, NJ). An antibiogram was conducted using the disk-diffusion method to detect meropenem-resistant gram-negative bacteria. Carbapenemase confirmation and classification of isolates resistant or intermediately resistant to meropenem were performed using the NMIC500 CPO kit on the Phoenix M50 system. **Results:** Among 599 isolates of gram-negative bacilli, 108 isolates were resistant or intermediately resistant to carbapenem (meropenem). Of these108 isolates, 107 (99.1%) were resistant due to the carbapenemase-producing mechanism. The proportions of resistant or intermediately resistant isolates to carbapenem were as follows: 73.8% for *Acinetobacter baumannii*, 26.4% for *Klebsiella pneumoniae*, 25.9% for *Pseudomonas aeruginosa*, and 2.8% for *Escherichia coli*. Class D carbapenemase accounted for the highest proportion, with 53 (49.5%) of 107 isolates, followed by class B with 31 isolates (29%), and class A with the lowest proportion of 2 isolates (1.9%). Also, 44.4% of *Acinetobacter baumannii* isolates and 74.4% of *Klebsiella pneumoniae* isolates produced class D carbapenemase. **Conclusions:** Gram-negative bacilli are resistant to carbapenem primarily due to the carbapenemase-secreting mechanism. D-class carbapenemase accounted for the highest percentage, followed by B-class type, and A-class carbapenemase in gram-negative bacilli.

